# Changes in Spinal Neural Circuit Plasticity in a Rat Sciatic Nerve Transection Model

**DOI:** 10.1002/brb3.71256

**Published:** 2026-02-05

**Authors:** Katsuyuki Konishi, Toru Iwahashi, Taisuke Kasuya, Toshiki Shimada, Yoshiaki Yoshimura, Atsushi Kamata, Mai Konishi, Mingyuan Wang, Arisa Kazui, Ryoya Shiode, Satoshi Miyamura, Kunihiro Oka, Seiji Okada, Hiroyuki Tanaka

**Affiliations:** ^1^ Department of Orthopaedic Surgery Osaka University Graduate School of Medicine Suita Osaka Japan; ^2^ Department of Sports Medical Science Osaka University Graduate School of Medicine Suita Osaka Japan

**Keywords:** cholinergic interneurons, corticospinal tract, plasticity changes, sciatic nerve transection

## Abstract

**Background and Aim:**

Changes in neural plasticity crucially modulate functional recovery after central nerve injury. To describe morphological peripheral nerve injury‐related changes, we comprehensively elucidated post‐sciatic nerve transection (post‐SNT) plasticity changes in the lumbar spinal corticospinal tract (CST), motor neurons, and cholinergic interneurons (CINs).

**Methods:**

In a rat SNT model, we compared post‐SNT changes in lumbar spinal cord plasticity at 2, 4, and 6 weeks with those in the Sham group. Using neural tracers and immunohistochemistry, we labeled and analyzed the CST axonal number and volume, motor neuron cell‐body volume and synaptic inputs, and cholinergic interneuron (CIN) (medial and lateral, based on the distance from the central canal) number and synaptic inputs.

**Results:**

Compared to the Sham group, the SNT groups showed no significant post‐SNT changes in CST axonal number and volume. Motor neuron cell‐body volume decreased by 29% at 6 weeks post‐SNT, and vesicular glutamate transporter 1 (vGlut1) and vesicular acetylcholine transporter (vAchT) synaptic inputs decreased by 82–93% and 27–42%, respectively, from 2 weeks post‐SNT onward. From 2 weeks post‐SNT onward, cell numbers were maintained, although vGlut1 synaptic inputs increased by 98%–68% in lateral CINs. At 6 weeks post‐SNT, a 44% reduction in cell numbers and a simultaneous 107% increase in vGlut1 synaptic inputs were noted in medial CINs.

**Conclusion:**

Following SNT, although CST axonal numbers remained unchanged, plasticity changes were observed in motor neurons and in CINs. CINs exhibited distinct plasticity changes depending on their localization.

## Graphical abstract

Following sciatic nerve transection (SNT), corticospinal tract (CST) labeling in the lumbar gray matter remained unchanged, whereas motor neurons showed soma atrophy with reduced perisomatic vesicular glutamate transporter 1 (vGlut1) and vesicular acetylcholine transporter (vAchT) inputs. In contrast, cholinergic interneurons (CINs) exhibited localization‐dependent plasticity: lateral CINs showed an early increase in vGlut1 inputs without cell loss, while medial CINs showed a delayed increase in vGlut1 inputs accompanied by reduced cell number.

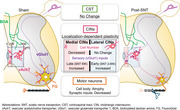

## 1 Introduction

Plasticity changes contribute to functional recovery after central nerve injury (Loy and Bareyre 2019). Clinical interventions promoting plasticity—such as early rehabilitation and transcranial magnetic stimulation—have proven effective (Liew et al. 2014). In contrast, axonal regeneration constitutes an important recovery aspect in peripheral nerve injury. With mild axonal injury, conservative treatment is preferred, but if axonal transection occurs, surgical interventions become necessary. Despite these treatments, satisfactory functional recovery is frequently not achieved (Grinsell and Keating 2014).

Previous studies focused on increasing injured motor neuron survival or controlling Wallerian degeneration (Modrak et al. 2020). However, limited literature exists regarding plasticity changes following peripheral nerve injury, with studies documenting only reduced synaptic inputs to motoneurons and decreased cell body volume (Navarro et al. 2007). Reduced synaptic inputs (so‐called synaptic stripping) are notably observed at vesicular glutamate transporter 1 (vGlut1) synapses on proprioceptive and mechanosensitive afferent fibers. In compression injuries, vGlut1 synaptic inputs recover with nerve regeneration. In transection injuries, even with successful nerve suturing, vGlut1 synaptic inputs remain permanently reduced, causing functional deficits like stretch reflex loss (Bullinger et al. 2011; Alvarez et al. 2011). Despite attempts to restore reduced partial vGlut1 synaptic inputs experimentally, this recovery does not always improve function (Rotterman et al. 2024). Thus, effective recovery may require reconstruction of both injured motor neurons and higher‐level neural circuits.

We used a rat sciatic nerve transection (SNT) model to investigate lumbar spinal cord plasticity changes using tracer labeling and immunostaining. We focused on the corticospinal tract (CST), which shows plasticity after central nerve injury, and cholinergic interneurons (CINs), which provide vesicular acetylcholine transporter (vAchT) synaptic inputs to motor neurons. The CST forms compensatory neural circuits after spinal cord injury by increasing collateral sprouting (Loy and Bareyre 2019). While evidence shows plasticity changes in the motor cortex following peripheral nerve injury (Zhang et al. 2015), changes in CST axons at the spinal cord level remain unclear. Additionally, vAchT synapses formed by CINs play a role in enhancing and modulating motor output through cholinergic inputs to motor neurons (Bertrand and Cazalets 2011; Zagoraiou et al. 2009). In spinal cord injury models, CIN cell numbers decrease (Jiang et al. 2018); reduced vAchT synaptic inputs to motor neurons have been shown to contribute to motor dysfunction (Skup et al. 2012; Kapitza et al. 2012). Furthermore, prolonged electrical stimulation of sensory nerves has been reported to induce not only increased vGlut1 synaptic inputs but also simultaneously induce increased vAchT synaptic inputs to motor neurons, contributing to enhanced reflex responses on the stimulated side (Gajewska‐Woźniak et al. 2016). This suggests an association between sensory nerves and higher neural circuits in motor function. Regarding peripheral nerve injury, vAchT synaptic inputs from CINs to motor neurons undergo synapse stripping similar to vGlut1 inputs (Alvarez et al. 2020; Salvany et al. 2021). However, plasticity changes in CINs themselves or in synaptic inputs to CINs have not been evaluated.

In this study, we aimed to perform morphological analysis of motor neurons, CST, and CINs following SNT to clarify synaptic and cellular plasticity changes in the spinal cord after peripheral nerve injury.

## 2 Materials and Methods

### 2.1 Experimental Animals and SNT Surgery

**FIGURE 1:**
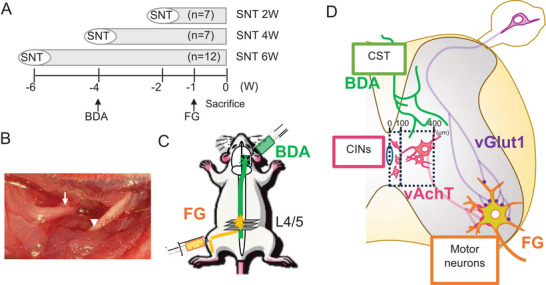
Experimental procedure. (A) Grouping of experimental animals; (B) SNT surgery (Arrowhead: proximal stump; Arrow: distal stump); (C) Schematic representation of neural tracing; and (D) Schematic of immunostained lumbar spinal cord. **Abbreviation**: SNT, sciatic nerve transection.

Thirty‐six 5‐week‐old female Wistar rats (body weight 180–220 g, Charles River Laboratories Japan) were used. The animals were assigned to Sham group (*n* = 10) and three post‐operative groups (Figure 1A): SNT 2 W (n = 7), SNT 4 W (*n* = 7), and SNT 6 W (*n* = 12). Anesthesia was induced via subcutaneous triple anesthetic injection. For SNT, as described previously (Wu et al. 2013), the left sciatic nerve was exposed, isolated, and transected above the adductor muscle. To prevent regeneration, the proximal stump was ligated, inverted, embedded in the biceps femoris muscle, and secured to the fascia. The distal stump was sutured to the adductor muscle (Figure 1B). The sham group underwent only exposure and isolation of the sciatic nerve.

We selected 5‐week‐old animals to ensure uniformity in surgical procedures and housing management for morphological evaluation up to 6 weeks post‐transection. Female animals exhibit less autotomy than do males following traumatic stress (Weber et al. 1993). Since this study focused on plasticity changes related to sensory inputs as the primary outcome, we used only female animals to minimize secondary effects caused by autotomy. No autotomy was observed in any animal during the observation period.

The animals were housed in a facility maintained at 23 ± 1.5°C, 45 ± 15% humidity, with a 12‐hour light/dark cycle (light/dark: 12 h/12 h). Standard chow and water were provided ad libitum. All groups were housed 2–3 animals per cage both before and after surgery, ensuring identical housing conditions throughout the study.

### 2.2 Neural Tracing

Anterograde tracing was performed four weeks before perfusion fixation (Figure 1C) (Liu et al. 2008; Starkey et al. 2012). Under anesthesia, animals were secured in a stereotaxic frame, and 10% biotinylated dextran amine (BDA, Invitrogen, Cat# D1956) was injected into the hindlimb motor cortex of the right cerebral hemisphere (anterior to bregma (AP) = −1.0 mm; lateral to bregma (ML) = +2.0 mm, and AP = −2.0 mm; ML = +2.0 mm, both at 1.5‐mm depth). Injections were performed using a stainless steel needle connected to a NanoFil syringe (World Precision Instruments, Sarasota, FL, USA), and 500 nL was injected at each site at a rate of 100 nL/min using a syringe pump. After each injection, the needle was left in place for 5 min (Starkey et al. 2012; DiGiovanna et al. 2016; Swanson 2018).

Retrograde tracing was performed using 4% FluoroGold (FG, Biotium, Cat# 80023) one week before perfusion (Hsu et al. 2017). Thereafter, 2 µL FG was manually injected into each side of the sciatic nerve over 1 min. On the transected side, injection was administered from the proximal nerve stump; on the intact side, it was performed at the sciatic nerve above the adductor muscle.

### 2.3 Immunohistochemical Staining

All 11‐week‐old animals were euthanized by inducing pneumothorax. Subsequently, cardiac perfusion with 1× PBS followed by chilled 4% paraformaldehyde (PFA) was performed. The L4–5 lumbar spinal cord segments were dissected, post‐fixed overnight in 4% PFA, and dehydrated for 2 days in 25% sucrose solution. Using a cryostat, three 50‐µm‐thick transverse sections were cut at 1‐mm intervals from each specimen.

First, the sections were washed with 1× PBS, then transferred to tubes containing 150 µL blocking solution (5% normal goat serum, 0.4% Triton‐X, 1× PBS) for blocking treatment. Next, the sections were incubated overnight at 4°C in 150 µL blocking solution containing anti‐rabbit vAchT (1:300; Synaptic Systems, Cat# 139103) and anti‐guinea pig vGlut1 (1:500; Millipore, Cat# AB5905). Then, the sections were incubated for 2 h at room temperature in 150 µL blocking solution containing goat‐anti‐rabbit Alexa Fluor Plus 594 (1:300; Invitrogen, Cat# A32740), goat‐anti‐guinea pig Alexa Fluor 647 (1:300; Invitrogen, Cat# A‐21450), and Streptavidin‐Dylight488 (1:150; Vector Laboratories, Cat# SA‐5488) for BDA labeling. Finally, the sections were cleared for 1 h using Rapi‐Clear 1.47 (SunJin Lab, Cat# RC147001), mounted on slide glasses, and covered with a cover slip using Rapi‐Clear 1.47 as the mounting medium.

All images were acquired using a Nikon AX‐R confocal microscope with a 40X objective lens (Nikon Lambda S 40XC Sil, 1024 × 1024 pixels, X1.0 zoom, 12‐bit) under identical conditions adjusted for each analysis (Figure 1D).

Considering CST analysis, 20 µm‐thick sections were acquired (z resolution 0.5 µm × 40 stacks). In regard to motor neuron analysis, images were adjusted to include the ventral horn; 40 µm‐thick sections were acquired (z resolution 1 µm × 40 stacks). With respect to CIN analysis, images were oriented to ensure that the central canal was positioned at the center of the image; 40 µm‐thick sections were acquired (z resolution 1 µm × 40 stacks).

### 2.4 Image Analysis

#### 2.4.1 Quantitative Analysis of CST

The standardized procedure using NIS‐Elements was performed (Liu et al. 2008). The boundary between the gray matter (as visualized by the tissue's autofluorescence) and the dorsal column was delineated using the freehand Region of Interest (ROI) tool, thereby creating separate ROIs for the gray matter and the dorsal column. BDA‐labeled axons above a predefined brightness threshold (smooth 0.43 µm, threshold (intensity) >700) were reconstructed as 3D axonal structures.

After reconstruction, the following analyses were performed using General Analysis 3 included in NIS‐Elements. The number of axons labeled in the dorsal column was counted from the central frame of the image obtained within the dorsal column ROI, considering only signals above a predefined brightness threshold. The number of axons extending from the dorsal column into gray matter was determined by counting the 3D axonal structures present on the boundary between the gray matter ROI and the dorsal column ROI. The volume of corticospinal axons within the gray matter was measured as the total volume of the 3D axonal structures in the gray matter ROI. For each animal, these measurements were obtained from three consecutive images, and the mean value was calculated.

#### 2.4.2 Quantitative Analysis of Motor Neurons

The standardized procedure using IMARIS 9.9.1 (Oxford Instruments plc) was employed (Arvidsson et al. 1997). Using the “Surface” function, vAchT labeling above a specified brightness threshold (surfaces smooth 0.5 µm, threshold (background subtraction) >150) was reconstructed as synaptic structures, and the brightness within these structures was masked to zero. Subsequently, the cytoplasm with brightness above a set threshold (surfaces smooth 5 µm, threshold (absolute intensity) >350) was similarly reconstructed with the “Surface” function to define motor neuronal cell bodies. In addition, structures that exhibited both vAchT and vGlut1 labeling above the predetermined brightness and voxel thresholds (Spot Diameter 3 µm, Threshold (intensity Max) >3500 (for vAchT synapse), Spot Diameter 3 µm, Threshold (intensity Max) >500, Quality Filter >150 (for vGlut1 synapse)) were detected using the “Spot” function and labeled as vAchT and vGlut1 synapses.

In this study, three‐dimensional extraction of the entire proximal dendritic tree as an ROI was technically difficult. Therefore, the perisomatic region extending from the cell body to the dendritic branch points was analyzed; only synapses contacting this perisomatic region were quantified.

To evaluate the volume of motor neuron cell bodies, three FG‐labeled cell bodies were selected per section; their average volume and average surface area were calculated. In these three cells, the number of vAchT and vGlut1 synaptic inputs detected via the “Spot” function was counted; an average value per cell was computed. Synaptic input counts were normalized by the surface area of each cell to obtain synaptic density; mean synaptic density values were calculated. In each animal, these measurements were performed on a total of 9 motor neurons from three consecutive sections; the mean value was used for analysis.

#### 2.4.3 Quantitative Analysis of CINs

vAchT can be used as a synapse‐specific marker for CINs, but it can also label the perinuclear region of cell bodies (Arvidsson et al. 1997). For analysis, an area extending 400‐µm laterally from the central canal toward the SNT‐treated side was evaluated. CINs were subdivided into medial CINs (within 100 µm from the central canal) and lateral CINs (between 100 and 400 µm) and evaluated separately.

The procedure similar to that for motor neurons was utilized with IMARIS 9.9.1 (Arvidsson et al. 1997). First, vAchT labeling above a certain brightness threshold (surfaces smooth 0.5 µm, Threshold (background subtraction) >150) was reconstructed as synaptic structures using the “Surface” function, and the brightness within these structures was masked to zero. Then, structures were defined as CINs when the cytoplasm exceeded the brightness threshold (surfaces smooth 1.0 µm, Threshold (absolute intensity) >250, Voxels Filter >10) and contained an unlabeled nucleus‐like low‐intensity region with regard to vAchT. Signals meeting the predetermined brightness thresholds (Spot Diameter 3 µm, Threshold (intensity Max) >3500 (for vAchT synapse), Spot Diameter 3 µm, Threshold (intensity Max) >500, Quality Filter >150 (for vGlut1 synapse)) were detected using the “Spot” function and labeled as vAchT and vGlut1 synapses.

In each animal, medial and lateral CINs were counted from three consecutive images. Furthermore, the total cell count from three sections was used as the CIN cell number. Since three‐dimensional reconstruction of CIN cell bodies alone was difficult in this study, cell body size was evaluated based on previous reports (Jensen et al. 2020) by selecting the section with the maximum nuclear cross‐section and measuring the long and short diameters of the cell body using the “Measurement Points” function in IMARIS 9.9.1. As with motor neurons, the perisomatic region extending from the cell body to the dendritic branch points was analyzed, and only synapses contacting this perisomatic region were quantified. In all identified CINs, cell numbers, long and short diameters of cell bodies, and vAchT and vGlut1 synaptic input counts detected by the “Spot” function were recorded, and the mean synaptic input number per cell was calculated from three sections for each animal.

### 2.5 Statistical Analysis

One‐way analysis of variance (ANOVA) followed by Dunnett's post hoc test was used for multiple comparisons of the measurement results, with the Sham group as the control, using two‐tailed tests. All statistical analyses were performed using Prism 10 software (GraphPad Software). The level of statistical significance was set at *p* < 0.05.

The n in statistical analyses represents the number of animals, not the number of cells or sections. In regard to CST, motor neurons, and CINs, three consecutive sections were analyzed per animal. In addition, the mean value from three images was used; for motor neurons, the mean value from a total of 9 cells was used; and for CINs, the mean value from all observable CINs was calculated. The mean value for each animal was then used for statistical analysis.

## 3 Results

### 3.1 No Significant Post‐SNT Plasticity Changes in CST Axon Number and Axonal Volume

**FIGURE 2:**
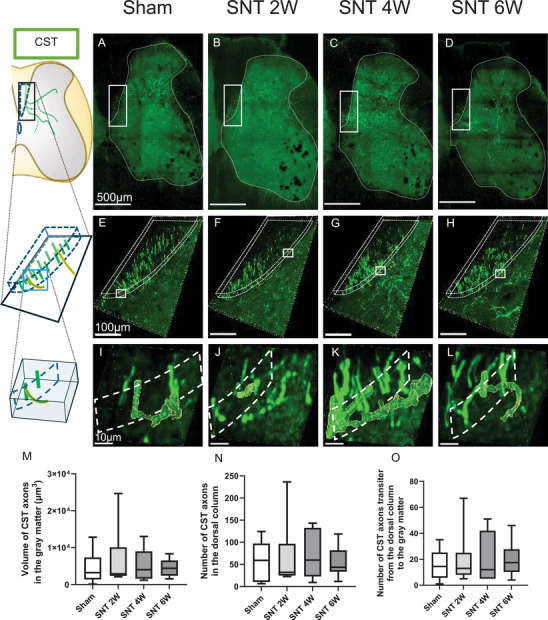
No significant post‐SNT plastic changes in CST axon number and axonal volume. (A–D) Stained images of the lumbar spinal cord on the SNT side; (E–H) Magnified images of the dorsal column (regions outlined in solid lines in A–D); (I–L) CST axons transition from the dorsal column to the gray matter (regions outlined in solid lines in E–H); (M) CST axonal volume in the gray matter, (N) Number of CST axons in the dorsal column; and (O) Number of CST axons transition from the dorsal column to the gray matter. Scale bars: (A–D) 500 µm; (E–H) 100 µm; (I–L) 10 µm. Sham group, *n* = 10; SNT 2 W group, *n* = 7; SNT 4 W group, *n* = 7; SNT 6 W group, *n* = 12. Dunnett's test. Box plots display the 25th to 75th percentiles, with the median shown as the central line and whiskers indicating the minimum and maximum values. **Abbreviation**: SNT, sciatic nerve transection.

In rodents, the CST is the primary descending pathway that projects from the cerebral cortex through the dorsal column to the gray matter and plays a crucial role in motor function. To assess post‐SNT morphological changes in CST axons, anterograde BDA tracing was performed. Observations and quantitative evaluations of the gray matter (Figures 2A–D), in the dorsal column (Figures 2E–H), and at the transition from the dorsal column to the gray matter (Figures 2I–L) were carried out. The results showed that the axonal volume of CST axons within the gray matter did not differ significantly between any SNT group and the Sham group (Figure 2M). Similarly, there were no significant changes in the number of descending CST axons in the dorsal column (Figure 2N) or in the number of CST axons entering the gray matter from the dorsal column (Figure 2O). These findings suggest that, at least at the lumbar spinal cord level, there were no apparent changes in the CST axonal number and volume up to 6 weeks post‐SNT.

### 3.2 Post‐SNT Reduction in Motor Neuron Cell‐body Volume and Synaptic Inputs

**FIGURE 3:**
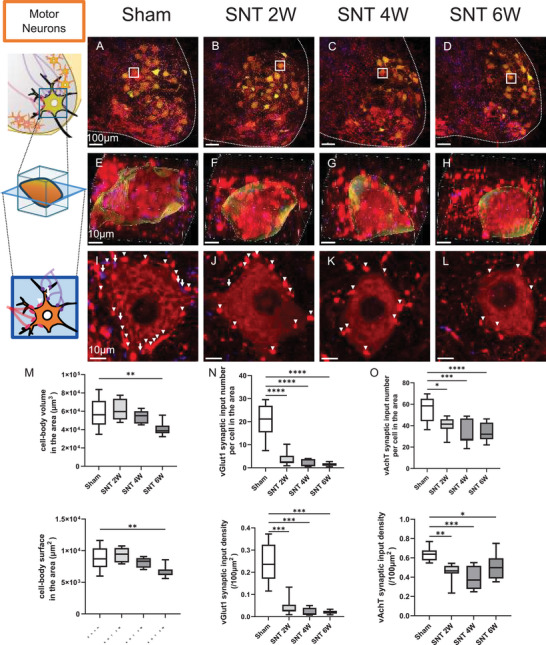
Post‐SNT reduction in the volume and synaptic input of the motor neuron cell body. (A–D) Stained images of the ventral lumbar spinal cord on the SNT side; (E–H) Enlarged 3D images of motor neurons (regions outlined in solid lines in A–D); (I–L) Motor neuron slices (Arrow: vGlut1 synaptic input; Arrowhead: vAchT synaptic input); (M) Average of motor neuron cell body volume; (N) Number of vGlut1 synaptic inputs to motor neurons per cell; (O) Number of vAchT synaptic inputs to motor neurons per cell. Scale bars: (A–D) 100 µm; (E–H) 10 µm; (I–L) 10 µm, (P) Average of motor neuron cell body surface area; (Q) Density of vGlut1 synaptic inputs to motor neurons per cell; and (R) Density of vAchT synaptic inputs to motor neurons per cell. Sham group, *n* = 10; SNT 2 W group, *n* = 7; SNT 4 W group, *n* = 7; SNT 6 W group, *n* = 12. (Dunnett's test ****p < 0.001, ***p < 0.005, **p < 0.01, and *p < 0.05). Box plots display the 25th to 75th percentiles, with the median shown as the central line and whiskers indicating the minimum and maximum values. **Abbreviation**: SNT, sciatic nerve transection.

Next, we evaluated changes in motor neurons that were directly affected by SNT. Retrograde tracing using FG was performed to label the motor neurons within the sciatic nerve (Figures 3A–D), and the changes in cell‐body volume (Figures 3E–H) and synaptic input number (Figures 3I–L) were analyzed using immunostaining. The cell‐body volume in the SNT 6 W group exhibited a significant 29% reduction compared with the Sham group (Figure 3M). Regarding synaptic inputs, the number of sensory nerve (vGlut1) synaptic inputs showed a significant time‐dependent reduction (SNT 2W: 82%, 4W: 91%, 6W: 93%; Figure 3N). Moreover, the number of cholinergic (vAchT) synaptic inputs from CINs decreased (SNT 2W: 27%, 4W: 42%, 6W: 39%; Figure 3O).

Additionally, the surface area of motor neuron cell bodies showed a significant 18% reduction in the SNT 6 W group compared with the Sham group (Figure 3P). When synaptic input counts were normalized by surface area to evaluate perisomatic synaptic density, vGlut1 synaptic density showed reductions of 82% at 2 W, 91% at 4 W, and 92% at 6 W post‐SNT (Figure 3Q), while vAchT synaptic density showed reductions of 29% at 2 W, 39% at 4 W, and 19% at 6 W (Figure 3R). Both vGlut1 and vAchT synaptic densities showed similar reduction trends as the respective input counts.

These results suggest that motor neurons that were directly injured by SNT underwent atrophy and synaptic stripping.

### 3.3 CINs Within 400 µm of the central Canal Exhibited Post‐SNT Increase in vGlut1 and vAchT Synaptic Inputs

**FIGURE 4:**
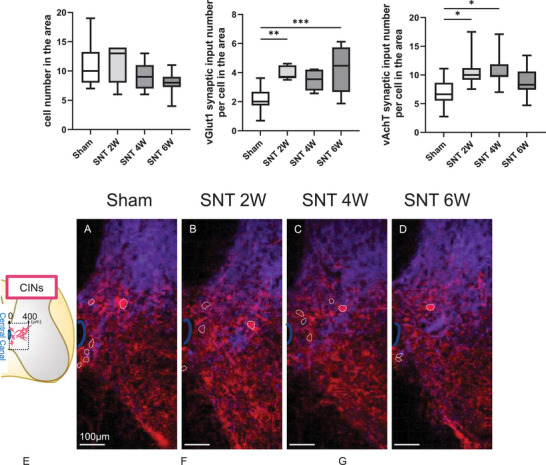
CINs within 400 µm of the central canal exhibited increased vGlut1 and vAchT synaptic input after SNT. (A–D) Stained images of CINs located within 400 µm of the central canal; (E) Cell numbers of CINs in the area; (F) Number of vGlut1 synaptic inputs to CINs per cell; and (G) Number of vAchT synaptic inputs to CINs per cell. Scale bars: (A–D) 100 µm. Sham group, *n* = 10; SNT 2 W group, *n* = 7; SNT 4 W group, *n* = 7; SNT 6 W group, *n* = 12. (Dunnett's test, ***p < 0.005, **p < 0.01, and *p < 0.05). Box plots display the 25th to 75th percentiles, with the median shown as the central line and whiskers indicating the minimum and maximum values. **Abbreviation**: SNT, sciatic nerve transection.

To examine the possibility that compensatory neural circuits form after the stripping of synaptic inputs to motor neurons, we evaluated the plasticity changes in CINs located near the central canal. The normal structure and function of the cholinergic circuits that are formed by CINs in the spinal cord remain largely unclear. The sensory nerve (vGlut1) synaptic input to CINs is inherently sparse (Zagoraiou et al. 2009). In contrast, the output from CINs via vAchT synapses not only connects to motor neurons (Tillakaratne et al. 2014) but also projects to CINs in adjacent segments and to interneurons that constitute central pattern generators, thereby regulating locomotor functions as intrinsic spinal circuits (Sherriff and Henderson 1994; Finkel et al. 2014; Anglister et al. 2017). In this study, we focused on CINs located within 400 µm of the central canal (Figures 4A–D) and performed quantitative analyses of cell numbers as well as vGlut1 and vAchT synaptic input numbers. Despite a trend toward a reduction in cell number in the SNT 6 W group compared with the Sham group, the difference was not significant (Figure 4E, *p* = 0.092). In contrast, the number of sensory (vGlut1) synaptic inputs was significantly increased in both the SNT 2 W and SNT 6 W groups, with the SNT 4 W group showing an increasing trend (Figure 4F, *p* = 0.054). Furthermore, the number of cholinergic (vAchT) synaptic inputs from CINs significantly increased in the SNT 2 and 4 W groups; however, this difference was non‐significant in the SNT 6 W group (Figure 4G).

Additionally, based on the acquired images and with reference to the report by Tillakaratne et al. (2014), CINs located 100–400 µm from the central canal were defined as lateral CINs, and those within 100 µm of the central canal were defined as medial CINs; cell body size was measured and evaluated. In the Sham group, lateral CINs had a long diameter of 28.08 µm and a short diameter of 20.72 µm, while medial CINs had a long diameter of 20.39 µm and a short diameter of 14.63 µm; both showed elliptical cell bodies. However, lateral CINs were larger in both long and short diameters compared with medial CINs. No significant differences in cell body long or short diameters were observed between the sham group and any SNT group for either lateral or medial CINs (Supplementary Table S1). Subsequently, cell numbers and synaptic inputs were analyzed separately for medial and lateral CINs.

### 3.4 Lateral CINs Showed Post‐SNT Increase in vGlut1 Synaptic Inputs, Without Change in Cell Number

**FIGURE 5:**
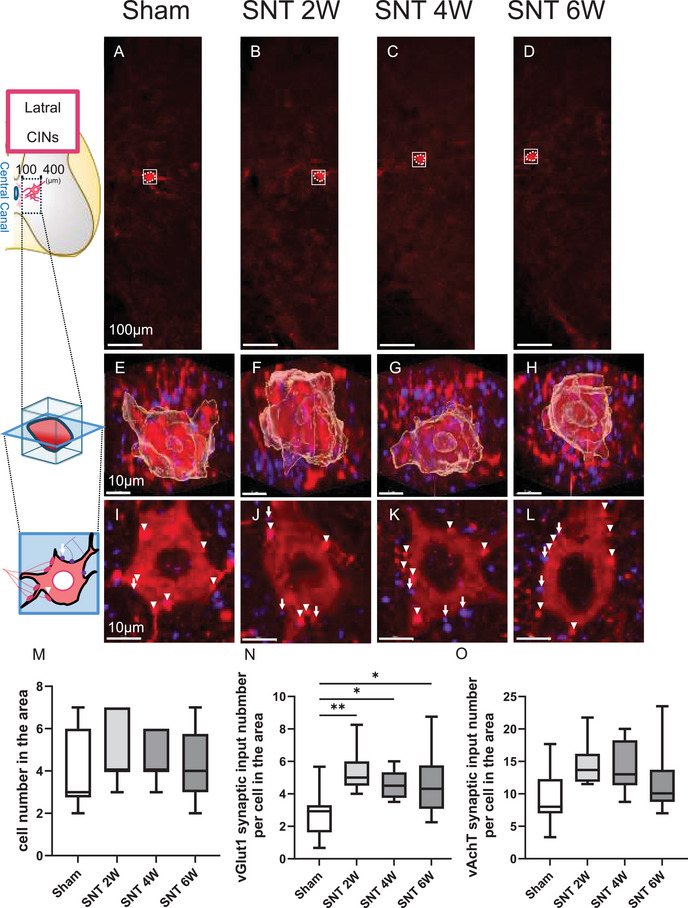
Lateral CINs showed a post‐SNT increase in vGlut1 synaptic inputs, without a change in cell number. (A–D) Stained images of lateral CINs on the SNT side; (E–H) Enlarged 3D images of lateral CINs (regions outlined in solid lines in A–D); and (I–L) Slices of interneurons (Arrow: vGlut1 synaptic input; Arrowhead: vAchT synaptic input); (M) Cell numbers of lateral CINs; (N) Number of vGlut1 synaptic inputs to lateral CINs per cell; and (O) Number of vAchT synaptic inputs to lateral CINs per cell. Scale bars: (A–D) 100 µm; (E–H) 10 µm; (I–L) 10 µm. Sham group, *n* = 10; SNT 2 W group, *n* = 7; SNT 4 W group, *n* = 7; SNT 6 W group, *n* = 12. (Dunnett's test **p < 0.01 and *p < 0.05). Box plots display the 25th to 75th percentiles, with the median shown as the central line and whiskers indicating the minimum and maximum values. **Abbreviation**: SNT, sciatic nerve transection.

Lateral CINs include a subset of partition cells in the lower lumbar spinal cord (Barber et al. 1984), among which the subgroup located near the central canal provides cholinergic input to motor neurons via vAchT synapses (Zagoraiou et al. 2009; Skup et al. 2012). As with the previous CINs analyses, we quantified the number of lateral CINs (Figures 5A–D) and their synaptic inputs (Figures 5E–L). The results showed that the cell numbers of lateral CINs, which possess relatively larger cell bodies (Figures 5E–L), did not differ significantly between any SNT groups and the Sham group (Figure 5M). In contrast, the number of sensory (vGlut1) synaptic inputs significantly increased in all SNT groups compared with the Sham group (SNT 2W: 98%, 4W: 68%, 6W: 68%; Figure 5N). Although the number of cholinergic (vAchT) synaptic inputs from CINs tended to increase in the SNT 2 W (*p* = 0.051) and SNT 4 W (*p* = 0.094) groups, these differences were not statistically significant (Figure 5O). Notably, although sensory (vGlut1) synaptic inputs to motor neurons decreased after 2 weeks post‐SNT (Figure 3N), sensory inputs to the lateral CINs increased.

### 3.5 Medial CINs Exhibited a Post‐SNT Reduction in Cell Number and an Increase in vGlut1 Synaptic Inputs

**FIGURE 6:**
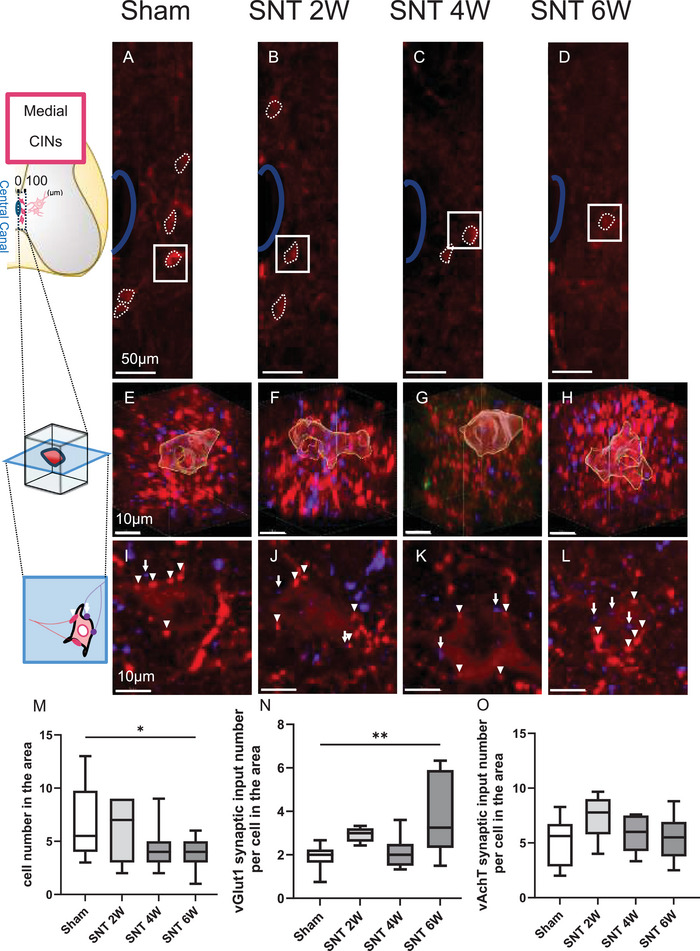
Medial CINs exhibited a post‐SNT reduction in cell number and an increase in vGlut1 synaptic input. (A–D) Stained images of medial CINs on the SNT side; (E–H) Enlarged 3D images of medial CINs (regions outlined in solid lines in A–D); (I–L) Slices of medial CINs (Arrow: vGlut1 synaptic input; Arrowhead: vAchT synaptic input); (M) Cell numbers of medial CINs; (N) Number of vGlut1 synaptic inputs to medial CINs per cell; and (O) Number of vAchT synaptic inputs to medial CINs per cell. Scale bars: (A–D) 100 µm; (E–H) 10 µm; (I–L) 10 µm. Sham group, *n* = 10; SNT 2 W group, *n* = 7; SNT 4 W group, *n* = 7; SNT 6 W group, *n* = 12. (Dunnett's test **p < 0.01 and *p < 0.05). Box plots display the 25th to 75th percentiles, with the median shown as the central line and whiskers indicating the minimum and maximum values. **Abbreviation**: SNT, sciatic nerve transection.

Medial CINs primarily include central canal cluster cells (CCC), as reported previously (Barber et al. 1984). Using a similar analysis as before, we quantified the number of medial CINs (Figure 6 A–D) and their synaptic inputs (Figures 6E–L).

The results showed that medial CINs, which are less intensely stained and have smaller cell bodies compared to lateral CINs (Figures 6E–L), showed a significant 44% reduction in cell number in the SNT 6 W group compared with the Sham group (Figure 6M), along with a significant 107% increase in sensory (vGlut1) synaptic inputs (Figure 6N). In the SNT 2 and 4 W groups, no significant changes in cell number or vGlut1 synaptic input numbers were observed. The number of cholinergic (vAchT) synaptic inputs from CINs showed a slight trend toward an increase in the SNT 2 W group (*p* = 0.117) compared with the Sham group, but no statistically significant differences were found in any SNT group (Figure 6O). Although lateral CINs did not exhibit any notable change in cell number (Figure 5M), medial CINs showed a decrease in cell number accompanied by an increase in sensory (vGlut1) synaptic inputs at 6 weeks post‐SNT, which suggests that distinct plasticity‐related changes occur in CINs depending on their localization after SNT.

## 4 Discussion

In this study, we examined morphological plasticity changes in CST, motor neurons, and CINs at the lumbar spinal cord level for up to 6 weeks post‐SNT. Our results showed no apparent changes in CST. However, plasticity changes in synaptic inputs and cells were observed in motor neurons and CINs. Specifically, motor neurons exhibited pronounced reductions in vGlut1 and vAchT synaptic inputs (synaptic stripping) and decreased cell‐body volume, while CINs showed different patterns of vGlut1 input increase and cell number changes depending on their localization.

Previous studies using intracortical microstimulation have shown that after peripheral nerve injury, affected motor areas become silent while adjacent regions compensate (Zhang et al. 2015; Socolovsky et al. 2017). Even after nerve regeneration, motor cortex reorganization remains incomplete. Combining rehabilitation with vagus nerve stimulation has been reported to improve mixed‐control areas and enhance motor function recovery (Meyers et al. 2019). However, CST plasticity at the spinal level remains unclear. Regarding spinal cord injury, CST collateral sprouting into the gray matter has been reported at 3 weeks post‐injury (Loy and Bareyre 2019, Filli and Schwab 2015; Bareyre et al. 2004). In contrast, our findings demonstrated that both axonal number and volume of CST fibers in the lumbar spinal cord remained unchanged for up to 6 weeks post‐SNT, without apparent induction in collateral sprouting. Unlike spinal cord injury, peripheral nerve injury does not directly damage the CST itself, suggesting that CST reorganization at the spinal level might be less likely to occur or may require longer observation periods. Additionally, CST changes following spinal cord injury involve not only collateral sprouting near the injury site but also alterations in intracortical connections (Ghosh et al. 2012) and the rubrospinal tract (Asboth et al. 2018). These pathway changes warrant investigation following peripheral nerve injury as well.

Although the animals used in this study were young (5‐week‐old), collateral sprouting following spinal cord injury has been reported in this age group (Schwab and Brösamle 1997), suggesting that plasticity changes can be examined. However, future studies should include age groups other than young animals.

Motor neurons exhibited post‐SNT plasticity changes, including a 29% reduction in cell‐body volume at 6 weeks, consistent with findings that reported a 7% reduction at 12 weeks post‐SNT (Rashidiani‐Rashidabadi et al. 2019). Sensory (vGlut1) synaptic inputs decreased by 82% at 2 weeks and remained reduced by 91%–93% thereafter. These results align with previous findings, which reported an 80%–90% reduction from 4 weeks after tibial nerve transection (Alvarez et al. 2011). Cholinergic (vAchT) synaptic inputs decreased by 27% at 2 weeks, leveling off at 39%–42% thereafter, and these results also align with previous findings (Salvany et al. 2019). Additionally, synaptic density normalized by surface area showed similar trends. The mechanisms underlying motor neuron atrophy and synaptic stripping remain unclear. Microglia have been implicated in initiating synaptic stripping (Alvarez et al. 2020; Salvany et al. 2021). Studies have shown that suppressing microglial function partially prevents vGlut1 synapse reduction and restores some inputs, but this does not necessarily improve function (Rotterman et al. 2024). This suggests that preserving pre‐injury spinal circuits is not always beneficial, and reconstructing or modifying alternative circuits might be more effective for functional recovery.

In this study, three‐dimensional extraction of the entire proximal dendritic tree was technically difficult. Therefore, following previous reports (Alvarez et al. 2011; Salvany et al. 2019), the analysis was limited to the perisomatic region extending from the cell body to the dendritic branch points. Three‐dimensional reconstruction analysis, including broader dendritic regions, remains a future challenge.

CINs displayed varying plasticity changes depending on their localization. Although no standardized definition exists, CINs in the gray matter can be classified into several groups based on their location (Barber et al. 1984; Phelps et al. 1990). In this study, lateral CINs had larger long and short diameters at the maximum nuclear cross‐section compared with medial CINs, as measured based on previous reports (Jensen et al. 2020), suggesting that they may represent morphologically distinct cell populations in terms of cell body size. In this study, three‐dimensional reconstruction of CIN cell bodies was difficult; moreover, synaptic density normalized by cell body surface area was not calculated. Therefore, changes in inputs to CINs were described as the number of synapses per cell. The long and short diameters at the maximum nuclear cross‐section, measured as indices of cell body size for both medial and lateral CINs, showed no significant differences between the sham and SNT groups, suggesting that surface area, which could affect synaptic density, was not markedly changed by SNT.

In lateral CINs, no significant change in cell number was observed, but sensory (vGlut1) synaptic inputs increased significantly from 2 weeks post‐SNT onward, coinciding with the period when vGlut1 inputs to motor neurons decreased. Although the absolute increase was limited to approximately 2–3 synapses per cell, vGlut1 inputs to CINs are inherently very sparse (Zagoraiou et al. 2009) (in this study, vGlut1 synaptic inputs in the Sham group were only 2.72 per cell in lateral CINs and 1.87 in medial CINs). Considering this, the relative increase cannot be dismissed; however, its functional significance requires further investigation. Previous studies have reported that reduced sensory (vGlut1) synaptic inputs to motor neurons after peripheral nerve injury are associated with decreased stretch reflexes and gait disturbances (Bullinger et al. 2011, Alvarez et al. 2011), and that microglia‐mediated vGlut1 synapse reorganization occurs not only at motor neurons but throughout the “motor circuit,” including surrounding circuits, resulting in relative enhancement of interneuron‐mediated pathways (Rotterman et al. 2019). The vGlut1 input changes observed in this study might potentially reflect redistribution of sensory inputs following SNT and could be related to functional changes in the future. However, functional conclusions cannot be drawn from the present data alone, and further investigation is needed.

Although not statistically significant, vAchT synaptic inputs to lateral CINs tended to increase at 2 and 4 weeks post‐SNT, while vAchT inputs to medial CINs showed an increasing trend at 2 weeks post‐SNT. CCC are longitudinally arranged along the central canal and provide cholinergic inputs to other CINs, contributing to short‐range intrinsic spinal segmental connectivity (Sherriff and Henderson 1994). The trend toward increased vAchT inputs observed post‐SNT may reflect transient enhancement of CIN connectivity in adjacent or injured spinal segments.

In medial CINs, vGlut1 synaptic inputs increased at 6 weeks post‐SNT, accompanied by a reduction in cell numbers. The relationship between these phenomena remains unclear, as no previous studies have directly linked increased synaptic input to interneuron loss. The delayed increase in vGlut1 inputs compared to lateral CINs may be due to differences in localization. Several mechanisms, including phagocytic cell death by activated microglia following spinal cord injury (Jiang et al. 2018) and degenerative cell death due to glutamate excitotoxicity (Kurabe et al. 2022), have been reported to contribute to interneuron loss. Additionally, CIN numbers in this study were assessed based on vAchT labeling, meaning the observed reduction may reflect decreased vAchT expression rather than actual cell loss. Central canal cluster cells (CCC), corresponding to medial CINs, are dual‐transmitter neurons that produce both acetylcholine and GABA (Gotts et al. 2016). In spinal cord injury, dual‐transmitter neurons can undergo neurotransmitter phenotype switching, leading to a reduction in neurotransmitter labeling (Bertels et al. 2022). Thus, in CCC, a shift in neurotransmitter phenotype may lead to decreased acetylcholine production and reduced vAchT expression.

This study has some limitations. First, this study used a model without spontaneous recovery to evaluate plasticity changes due to pure peripheral nerve injury. This is primarily a descriptive study based on morphological and anatomical observations, and quantitative behavioral or electrophysiological assessments were not performed on the same animals. Therefore, this study does not demonstrate a direct relationship between the observed synaptic and cellular plasticity changes and changes in motor function or gait patterns.

Second, this study used only 5‐week‐old young female animals, and the effects of age and sex were not assessed. Although 5‐week‐old animals have been shown to exhibit functional recovery and axonal repair after peripheral nerve injury that was not markedly different from adult animals (Kemp et al. 2015), the results of this study may not directly apply to adult or male animals. To clarify this point, future studies including male animals and different age groups are necessary.

Finally, three‐dimensional reconstruction of CIN cell body surfaces was difficult, and synaptic density normalized by surface area was not calculated for CINs. Additionally, we did not determine the cause of medial CIN reduction, and further investigation is needed to explore the relationship between increased vGlut1 inputs and cell loss. Combining labeling methods other than vAchT or CIN subtype‐specific genetic markers with three‐dimensional cell body reconstruction, synaptic inputs and morphological changes for each subtype could be evaluated more accurately.

## 5 Conclusion

Based on morphological analysis, this study demonstrated that SNT did not induce marked changes in CST axons but caused changes in synaptic inputs and cells in motor neurons and CINs. These findings describe changes in neural circuit connectivity and cells at the spinal level following peripheral nerve transection and provide fundamental information for investigating their functional significance through future combination with behavioral assessment and electrophysiological analysis.

## Author Contributions

K. K., T. I., and H. T. performed most of the experiments. K. K., T. I., T. K., T. S., Y. Y., A. K., and H. T. conceptualized the study and wrote the manuscript. T. I., M. K., M. W., A. K., R. S., S. M., K. O., S. O., and H. T. supported and helped write the manuscript. All the authors have read and approved the final submission of this paper.

## Funding

This work was supported in part by the Japan Society for the Promotion of Science (JSPS) KAKENHI (Grant Numbers T21K0902490).

## Ethics Statement

This study was approved by the Animal Experiment Ethics Committee of Osaka University.

## Conflicts of Interest

The authors declare no conflicts of interest.

## Supporting information




**Supplementary Table S1** Long and short diameters of CINs soma (mean ± SEM, µm). Sham group, *n* = 10; SNT 2 W group, *n* = 7; SNT 4 W group, *n* = 7; SNT 6 W group, *n* = 12. Dunnett's test. **Abbreviations**: SEM, standard error of the mean; SNT, sciatic nerve transection.

## Data Availability

All data analyzed in this study are available from the author (K.K.) and the corresponding author (T.I.) upon request.

## References

[brb371256-bib-0001] Alvarez, F. J. , T. M. Rotterman , E. T. Akhter , A. R. Lane , A. W. English , and T. C. Cope . 2020. “Synaptic Plasticity on Motoneurons After Axotomy: A Necessary Change in Paradigm.” Frontiers in Molecular Neuroscience 13: 68. 32425754 10.3389/fnmol.2020.00068 PMC7203341

[brb371256-bib-0002] Alvarez, F. J. , H. E. Titus‐Mitchell , K. L. Bullinger , M. Kraszpulski , P. Nardelli , and T. C. Cope . 2011. “Permanent Central Synaptic Disconnection of Proprioceptors After Nerve Injury and Regeneration. I. Loss of VGLUT1/Ia Synapses on Motoneurons.” Journal of Neurophysiology 106: 2450–2470. 21832035 10.1152/jn.01095.2010 PMC3214094

[brb371256-bib-0003] Anglister, L. , M. Cherniak , and A. Lev‐Tov . 2017. “Ascending Pathways That Mediate Cholinergic Modulation of Lumbar Motor Activity Supplement.” Journal of Neurochemistry 142, no. Suppl 2: 82–89. 28791705 10.1111/jnc.14065

[brb371256-bib-0004] Arvidsson, U. , M. Riedl , R. Elde , and B. Meister . 1997. “Vesicular Acetylcholine Transporter (VAChT) Protein: A Novel and Unique Marker for Cholinergic Neurons in the Central and Peripheral Nervous Systems.” Journal of Comparative Neurology 378: 454–467. 9034903

[brb371256-bib-0005] Asboth, L. , L. Friedli , J. Beauparlant , et al. 2018. “Cortico‐reticulo‐spinal Circuit Reorganization Enables Functional Recovery After Severe Spinal Cord Contusion.” Nature Neuroscience 21: 576–588. 29556028 10.1038/s41593-018-0093-5

[brb371256-bib-0006] Barber, R. P. , P. E. Phelps , C. R. Houser , G. D. Crawford , P. M. Salvaterra , and J. E. Vaughn . 1984. “The Morphology and Distribution of Neurons Containing Choline Acetyltransferase in the Adult Rat Spinal Cord: An Immunocytochemical Study.” Journal of Comparative Neurology 229: 329–346. 6389613 10.1002/cne.902290305

[brb371256-bib-0007] Bareyre, F. M. , M. Kerschensteiner , O. Raineteau , T. C. Mettenleiter , O. Weinmann , and M. E. Schwab . 2004. “The Injured Spinal Cord Spontaneously Forms a New Intraspinal Circuit in Adult Rats.” Nature Neuroscience 7: 269–277. 14966523 10.1038/nn1195

[brb371256-bib-0008] Bertels, H. , G. Vicente‐Ortiz , K. El Kanbi , and A. Takeoka . 2022. “Neurotransmitter Phenotype Switching by Spinal Excitatory Interneurons Regulates Locomotor Recovery After Spinal Cord Injury.” Nature Neuroscience 25: 617–629. 35524138 10.1038/s41593-022-01067-9 PMC9076533

[brb371256-bib-0009] Bertrand, S. S. , and J. R. Cazalets . 2011. “Cholinergic Partition Cells and Lamina X Neurons Induce a Muscarinic‐dependent Short‐Term Potentiation of Commissural Glutamatergic Inputs in Lumbar Motoneurons.” Front Neural Circuits 5: 15. 22069380 10.3389/fncir.2011.00015 PMC3208176

[brb371256-bib-0010] Bullinger, K. L. , P. Nardelli , M. J. Pinter , F. J. Alvarez , and T. C. Cope . 2011. “Permanent Central Synaptic Disconnection of Proprioceptors after Nerve Injury and Regeneration. II. Loss of Functional Connectivity With Motoneurons.” Journal of Neurophysiology 106: 2471–2485. 21832030 10.1152/jn.01097.2010 PMC3214087

[brb371256-bib-0011] DiGiovanna, J. , N. Dominici , L. Friedli , et al. 2016. “Engagement of the Rat Hindlimb Motor Cortex Across Natural Locomotor Behaviors.” Journal of Neuroscience 36: 10440–10455. 27707977 10.1523/JNEUROSCI.4343-15.2016 PMC6705591

[brb371256-bib-0012] Filli, L. , and M. E. Schwab . 2015. “Structural and Functional Reorganization of Propriospinal Connections Promotes Functional Recovery After Spinal Cord Injury.” Neural Regeneration Research 10: 509–513. 26170799 10.4103/1673-5374.155425 PMC4424731

[brb371256-bib-0013] Finkel, E. , A. Etlin , M. Cherniak , Y. Mor , A. Lev‐Tov , and L. Anglister . 2014. “Neuroanatomical Basis for Cholinergic Modulation of Locomotor Networks by Sacral Relay Neurons With Ascending Lumbar Projections.” Journal of Comparative Neurology 522: 3437–3455. 24752570 10.1002/cne.23613

[brb371256-bib-0014] Gajewska‐Woźniak, O. , K. Grycz , J. Czarkowska‐Bauch , and M. Skup . 2016. “Electrical Stimulation of Low‐threshold Proprioceptive Fibers in the Adult Rat Increases Density of Glutamatergic and Cholinergic Terminals on Ankle Extensor Alpha‐motoneurons.” PLoS ONE 11: e0161614. 27552219 10.1371/journal.pone.0161614 PMC4994941

[brb371256-bib-0015] Ghosh, A. , S. Peduzzi , M. Snyder , et al. 2012. “Heterogeneous Spine Loss in Layer 5 Cortical Neurons After Spinal Cord Injury.” Cerebral Cortex 22: 1309–1317. 21840844 10.1093/cercor/bhr191

[brb371256-bib-0016] Gotts, J. , L. Atkinson , Y. Yanagawa , J. Deuchars , and S. A. Deuchars . 2016. “Co‐expression of GAD67 and Choline Acetyltransferase in Neurons in the Mouse Spinal Cord: A Focus on Lamina X.” Brain Research 1646: 570–579. 27378584 10.1016/j.brainres.2016.07.001 PMC4986852

[brb371256-bib-0017] Grinsell, D. , and C. P. Keating . 2014. “Peripheral Nerve Reconstruction After Injury: A Review of Clinical and Experimental Therapies.” BioMed Research International 2014: 698256. 25276813 10.1155/2014/698256 PMC4167952

[brb371256-bib-0018] Hsu, S. T. , C. H. Yao , Y. M. Hsu , J. H. Lin , Y. H. Chen , and Y. S. Chen . 2017. “Effects of Taxol on Regeneration in a Rat Sciatic Nerve Transection Model.” Scientific Reports 7: 42280. 28181572 10.1038/srep42280 PMC5299405

[brb371256-bib-0019] Jensen, D. B. , S. Klingenberg , K. P. Dimintiyanova , J. Wienecke , and C. F. Meehan . 2020. “Intramuscular Botulinum Toxin A Injections Induce Central Changes to Axon Initial Segments and Cholinergic Boutons on Spinal Motoneurones in Rats.” Scientific Reports 10: 893. 31964988 10.1038/s41598-020-57699-z PMC6972769

[brb371256-bib-0020] Jiang, Y. Q. , A. Sarkar , A. Amer , and J. H. Martin . 2018. “Transneuronal Downregulation of the Premotor Cholinergic System After Corticospinal Tract Loss.” Journal of Neuroscience 38: 8329–8344. 30049887 10.1523/JNEUROSCI.3410-17.2018 PMC6158693

[brb371256-bib-0021] Kapitza, S. , B. Zörner , O. Weinmann , et al. 2012. “Tail Spasms in Rat Spinal Cord Injury: Changes in Interneuronal Connectivity.” Experimental Neurology 236: 179–189. 22569103 10.1016/j.expneurol.2012.04.023

[brb371256-bib-0022] Kemp, S. W. , C. D. Chiang , E. H. Liu , et al. 2015. “Characterization of Neuronal Death and Functional Deficits Following Nerve Injury During the Early Postnatal Developmental Period in Rats.” Developmental Neuroscience 37: 66–77. 25592862 10.1159/000368769

[brb371256-bib-0023] Kurabe, M. , M. Sasaki , K. Furutani , H. Furue , Y. Kamiya , and H. Baba . 2022. “Structural and Functional Properties of Spinal Dorsal Horn Neurons After Peripheral Nerve Injury Change Overtime via Astrocyte Activation.” Iscience 25: 105555. 36444301 10.1016/j.isci.2022.105555 PMC9700017

[brb371256-bib-0024] Liew, S. L. , E. Santarnecchi , E. R. Buch , and L. G. Cohen . 2014. “Non‐invasive Brain Stimulation in Neurorehabilitation: Local and Distant Effects for Motor Recovery.” Frontiers in Human Neuroscience 8: 378. 25018714 10.3389/fnhum.2014.00378 PMC4072967

[brb371256-bib-0025] Liu, Z. , Y. Li , X. Zhang , S. Savant‐Bhonsale , and M. Chopp . 2008. “Contralesional Axonal Remodeling of the Corticospinal System in Adult Rats After Stroke and Bone Marrow Stromal Cell Treatment.” Stroke; A Journal of Cerebral Circulation 39: 2571–2577. 10.1161/STROKEAHA.107.511659 PMC2593106 18617661

[brb371256-bib-0026] Loy, K. , and F. M. Bareyre . 2019. “Rehabilitation Following Spinal Cord Injury: How Animal Models Can Help Our Understanding of Exercise‐induced Neuroplasticity.” Neural Regeneration Research 14: 405–412. 30539806 10.4103/1673-5374.245951 PMC6334617

[brb371256-bib-0027] Meyers, E. C. , N. Kasliwal , B. R. Solorzano , et al. 2019. “Enhancing Plasticity in Central Networks Improves Motor and Sensory Recovery After Nerve Damage.” Nature Communications 10: 5782. 10.1038/s41467-019-13695-0 PMC6923364 31857587

[brb371256-bib-0028] Modrak, M. , M. A. H. Talukder , K. Gurgenashvili , M. Noble , and J. C. Elfar . 2020. “Peripheral Nerve Injury and Myelination: Potential Therapeutic Strategies.” Journal of Neuroscience Research 98: 780–795. 31608497 10.1002/jnr.24538 PMC7072007

[brb371256-bib-0029] Navarro, X. , M. Vivó , and A. Valero‐Cabré . 2007. “Neural Plasticity after Peripheral Nerve Injury and Regeneration.” Progress in Neurobiology 82: 163–201. 17643733 10.1016/j.pneurobio.2007.06.005

[brb371256-bib-0030] Phelps, P. E. , R. P. Barber , L. A. Brennan , V. M. Maines , P. M. Salvaterra , and J. E. Vaughn . 1990. “Embryonic Development of Four Different Subsets of Cholinergic Neurons in Rat Cervical Spinal Cord.” Journal of Comparative Neurology 291: 9–26. 2298930 10.1002/cne.902910103

[brb371256-bib-0031] Rashidiani‐Rashidabadi, A. , M. H. Heidari , E. Sajadi , et al. 2019. “Sciatic Nerve Injury Alters the Spatial Arrangement of Neurons and Glial Cells in the Anterior Horn of the Spinal Cord.” Neural Regeneration Research 14: 1833–1840. 31169202 10.4103/1673-5374.257539 PMC6585558

[brb371256-bib-0032] Rotterman, T. M. , E. T. Akhter , A. R. Lane , et al. 2019. “Spinal Motor Circuit Synaptic Plasticity after Peripheral Nerve Injury Depends on Microglia Activation and a CCR2 Mechanism.” Journal of Neuroscience 39: 3412–3433. 30833511 10.1523/JNEUROSCI.2945-17.2019 PMC6495126

[brb371256-bib-0033] Rotterman, T. M. , Z. Haley‐Johnson , T. S. Pottorf , et al. 2024. “Modulation of central Synapse Remodeling After Remote Peripheral Injuries by the CCL2‐CCR2 Axis and Microglia.” Cell reports 43: 113776. 38367237 10.1016/j.celrep.2024.113776 PMC10947500

[brb371256-bib-0034] Salvany, S. , A. Casanovas , L. Piedrafita , et al. 2021. “Microglial Recruitment and Mechanisms Involved in the Disruption of Afferent Synaptic Terminals on Spinal Cord Motor Neurons After Acute Peripheral Nerve Injury.” Glia 69: 1216–1240. 33386754 10.1002/glia.23959 PMC7986680

[brb371256-bib-0035] Salvany, S. , A. Casanovas , O. Tarabal , et al. 2019. “Localization and Dynamic Changes of Neuregulin‐1 at C‐type Synaptic Boutons in Association With Motor Neuron Injury and Repair.” FASEB Journal 33: 7833–7851. 30912977 10.1096/fj.201802329R

[brb371256-bib-0036] Schwab, M. E. , and C. Brösamle . 1997. “Regeneration of Lesioned Corticospinal Tract Fibers in the Adult Rat Spinal Cord Under Experimental Conditions.” Spinal Cord 35: 469–473. 9232753 10.1038/sj.sc.3100457

[brb371256-bib-0037] Sherriff, F. E. , and Z. Henderson . 1994. “A Cholinergic Propriospinal Innervation of the Rat Spinal Cord.” Brain Research 634: 150–154. 8156385 10.1016/0006-8993(94)90268-2

[brb371256-bib-0038] Skup, M. , O. Gajewska‐Wozniak , P. Grygielewicz , T. Mankovskaya , and J. Czarkowska‐Bauch . 2012. “Different Effects of Spinalization and Locomotor Training of Spinal Animals on Cholinergic Innervation of the Soleus and Tibialis Anterior Motoneurons.” European Journal of Neuroscience 36: 2679–2688. 22708650 10.1111/j.1460-9568.2012.08182.x

[brb371256-bib-0039] Socolovsky, M. , M. Malessy , D. Lopez , F. Guedes , and L. Flores . 2017. “Current Concepts in Plasticity and Nerve Transfers: Relationship Between Surgical Techniques and Outcomes.” Neurosurgical Focus [Electronic Resource] 42, no. 3: E13. 10.3171/2016.12.FOCUS16431 28245665

[brb371256-bib-0040] Starkey, M. L. , C. Bleul , B. Zörner , et al. 2012. “Back Seat Driving: Hindlimb Corticospinal Neurons Assume Forelimb Control Following Ischaemic Stroke.” Brain 135: 3265–3281. 23169918 10.1093/brain/aws270

[brb371256-bib-0041] Swanson, L. W. 2018. “Brain Maps 4.0‐Structure of the Rat Brain: an Open Access Atlas With Global Nervous System Nomenclature Ontology and Flatmaps.” Journal of Comparative Neurology 526: 935–943. 29277900 10.1002/cne.24381 PMC5851017

[brb371256-bib-0042] Tillakaratne, N. J. , P. Duru , H. Fujino , et al. 2014. “Identification of Interneurons Activated at Different Inclines During Treadmill Locomotion in Adult Rats.” Journal of Neuroscience Research 92: 1714–1722. 24975393 10.1002/jnr.23437

[brb371256-bib-0043] Weber, R. A. , W. H. Proctor , M. R. Warner , and C. N. Verheyden . 1993. “Autotomy and the Sciatic Functional Index.” Microsurgery 14: 323–327. 8332052 10.1002/micr.1920140507

[brb371256-bib-0044] Wu, P. , R. J. Spinner , Y. Gu , M. J. Yaszemski , A. J. Windebank , and H. Wang . 2013. “Delayed Repair of the Peripheral Nerve: A Novel Model in the Rat Sciatic Nerve.” Journal of Neuroscience Methods 214: 37–44. 23313757 10.1016/j.jneumeth.2013.01.003

[brb371256-bib-0045] Zagoraiou, L. , T. Akay , J. F. Martin , R. M. Brownstone , T. M. Jessell , and G. B. Miles . 2009. “A Cluster of Cholinergic Premotor Interneurons Modulates Mouse Locomotor Activity.” Neuron 64: 645–662. 20005822 10.1016/j.neuron.2009.10.017 PMC2891428

[brb371256-bib-0046] Zhang, J. , L. Chen , and Y. D. Gu . 2015. “Influence of Contralateral Homologous Cortices on Motor Cortical Reorganization After Brachial Plexus Injuries in Rats.” Neuroscience Letters 606: 18–23. 26314511 10.1016/j.neulet.2015.08.035

