# Supplementary material for: Changes in Spinal Neural Circuit Plasticity in a Rat Sciatic Nerve Transection Model
Source: Brain Behav. 2026 Feb 5;16(2):e71256. doi: 10.1002/brb3.71256 (PMC12876045; doi:10.1002/brb3.71256)
Supplement: Supplementary file 1 — Supplementary Table S1 Long and short diameters of CINs soma (mean ± SEM, µm). Sham group, n = 10; SNT 2 W group, n = 7; SNT 4 W group, n = 7; SNT 6 W group, n = 12. Dunnett's test. Abbreviations: SEM, standard error of the mean; SNT, sciatic nerve transection. [file BRB3-16-e71256-s001.docx]

|  | | Sham | SNT 2W | SNT 4W | SNT 6W |
| --- | --- | --- | --- | --- | --- |
| Lateral CINs | Major diameter | 28.08 ± 0.83 | 28.29 ± 1.16 | 27.85 ± 1.68 | 27.65 ± 1.04 |
|  | Minor diameter | 20.72 ± 0.63 | 20.73 ± 0.61 | 18.67 ± 0.46 | 20.49 ± 0.61 |
| Medial CINs | Major diameter | 20.39 ± 0.36 | 19.84 ± 0.40 | 20.33 ± 1.04 | 20.62 ± 0.61 |
|  | Minor diameter | 14.63 ± 0.27 | 14.92 ± 0.83 | 13.26 ± 0.58 | 14.56 ± 0.46 |

(mean ± SEM, Unit: μm)

Supplementary Table 1

Abbreviations: CINs, cholinergic interneurons; SEM, standard error of the mean; SNT, sciatic nerve transection
